# Cholangiocarcinoma Trends, Incidence, and Relative Survival in Khon Kaen, Thailand From 1989 Through 2013: A Population-Based Cancer Registry Study

**DOI:** 10.2188/jea.JE20180007

**Published:** 2019-05-05

**Authors:** Supot Kamsa-ard, Vor Luvira, Krittika Suwanrungruang, Siriporn Kamsa-ard, Varisara Luvira, Chalongpon Santong, Tharatip Srisuk, Ake Pugkhem, Vajarabhongsa Bhudhisawasdi, Chawalit Pairojkul

**Affiliations:** 1 Department of Epidemiology and Biostatistics, Faculty of Public Health, Khon Kaen University, Khon Kaen, Thailand; 2 ASEAN Cancer Epidemiology and Prevention Research Group, Khon Kaen University, Khon Kaen, Thailand; 3 Department of Surgery, Faculty of Medicine, Khon Kaen University, Khon Kaen, Thailand; 4 Cancer Unit, Srinagarind Hospital, Faculty of Medicine, Khon Kaen University, Khon Kaen, Thailand; 5 Department of Community Medicine, Faculty of Medicine, Khon Kaen University, Khon Kaen, Thailand; 6 Department of Pathology, Faculty of Medicine, Khon Kaen University, Khon Kaen, Thailand

**Keywords:** cholangiocarcinoma, trend, incidence, relative survival, cancer registry

## Abstract

**Background:**

Cholangiocarcinoma (CCA) is a common malignancy in northeastern Thailand. Over the last 4 decades, several policies have been implemented for its prevention, but there has been no update on the trends and relative survival (RS). Our aim was (a) to perform a statistical assessment of the incidence trends of CCA and project future trends, and (b) to estimate relative survival.

**Methods:**

All cases of CCA diagnosed from 1989 through 2013 were abstracted from the Khon Kaen Cancer Registry (KKCR). A jointpoint regression model was used to estimate the annual percentage change (APC) and to project future trends. We also calculated RS.

**Results:**

There were 11,711 cases of CCA. The incidence rate increased with an APC of 1.79% (95% confidence interval [CI], −0.2 to 3.8) from 1989 through 2002, and decreased with an APC of −6.09% (95% CI, −8.2 to −3.9) from 2002 through 2013. The projected incidence of CCA should stable over the next 10 years, albeit higher than the world rate. The respective 5-year RS for both sexes for age groups of 30–40, 41–45, 51–60, and 61–98 years was 22.3% (95% CI, 16.8–29.5), 14.3% (95% CI, 12.0–17.0), 8.6% (95% CI, 7.8–10.0), and 7.2% (95% CI, 6.4–8.0).

**Conclusion:**

The incidence rate of CCA has decreased since 2002, representing a real decline in the risk of CCA. The incidence of CCA is projected to stabilize by 2025. The survival of patients with CCA remains poor.

## INTRODUCTION

Cholangiocarcinoma (CCA) is a major cause of cancer mortality around the world.^1^ In Thailand, from 1988 through 2012, the respective age-standardized rate (ASR) of liver and bile duct cancer was between 53.4 and 94.8 per 100,000 for males and 18.5 and 39.4 per 100,000 for females. Among affected persons, CCA was the most common cell type, comprising between 82.0% and 89.0% of all detected primary liver cancers.^2^^–^^9^

Several risk factors for CCA have been investigated in Thailand, but *Opisthorchis viverrini* is most often implicated in the genesis of CCA.^10^^–^^13^ Since 1987, as a means of limiting the incidence of CCA, a number of government policies have been implemented to eradicate *O. viverrini* infection.^14^ Treatment of CCA has also been improved through (a) better and more timely use of diagnostic technology, (b) refined surgical techniques, and (c) increasing the number of surgeons. We reported the declining trend in the incidence of CCA over the past 20 years, and hypothesized this trend represents a real reduced risk for CCA.^15^ We attempted to use our reported data to simulate the predicted incidence of CCA in the future, but the reported incidence was not sufficiently stable. Therefore, we extended the period studied in order to (a) perform a statistical assessment of the incidence trends of CCA, (b) project future trends in the incidence of CCA, (c) evaluate the effectiveness of the control policies, and (d) evaluate whether the various treatment protocols have improved survival.

## MATERIAL AND METHODS

Data for the study were from the Khon Kaen Cancer Registry (KKCR), a population-based cancer registry for Khon Kaen Province in northeastern Thailand. The KKCR began in 1985. According to estimates from the census data of the National Statistical Office for 2012^16^ (available at URL: http://www.nso.go.th/), the KKCR contains data on 1.7 million patients comprising all cancer sites as per the International Agency for Research on Cancer (IARC) guidelines.^17^ The KKCR has a completeness rating of 97.0%.^18^

The sources of information for the cancer registry include databases from regional and community hospitals, pathology departments, and death certificates. The lag-time between diagnosis and reporting is less than 1 year, and only 3.2% of cases are based upon information from a death certificate only (DCO cases). All cases are encoded as per the International Classification of Diseases for Oncology, 3rd edition (ICD-O-3).^19^

### Case definitions

The database was searched for all patients with CCA tumors living in Khon Kaen Province between January 1, 1989 and December 31, 2013. CCA is an ICD-O-3 diagnosis, and only the cases with coding C22.1, C24.0, C24.8, and C24.9 (excluding C24.1, Ampulla of Vater) were included.^20^ Patients diagnosed from 1985 through 1988 were excluded because, at that time, registries were only just opening and the data lacked completeness, making the ASR unreliable.

### Statistical methods

Percentages were used to describe proportions of the categorical data. The mean (standard deviation [SD]) was used to describe the continuous variables. Incidence trends were assessed using the estimated annual percentage change (APC) of the world ASRs. The Jointpoint regression program (version 4.0.4; The Surveillance, Epidemiology, and End Results [SEER] program of the National Cancer Institute [NCI], Rockville, MD, USA.)^21^ was used to investigate the trends in the incidence rate of CCA, identify points where a significant change in the linear slope of the trends occurred, and the corresponding *P*-value and 95% confidence interval (CI) of the APC. We then determined the incidences of CCA in Khon Kaen Province from 1989 through 2013 and projected future trends from 2014 through 2030.^22^ A maximum of one point was allowed in each regression. A value of 0.01 was added to all of the years in the data series for the dependent variable where a zero value was observed in 1 or more years. To determine survival, we calculated the follow-up time from diagnosis to the last known vital status of each patient; this was obtained by linking records between the Mortality Registry of Thailand^23^ and the National Statistical Office^16^ (updated to December 31, 2016). Focusing on stage of disease and period of time, the survival analysis was estimated using the Kaplan-Meier survival curve, and the log-rank test was used for between group comparisons.^24^ Since the mean age at diagnosis of the neoplasms is high and other medical conditions may have influenced patient death, we also analyzed observed survival (OS) by stage of disease and period of diagnosis. In addition, we illustrated relative survival (RS), the ratio between observed survival (OS) and expected survival.^25^ RS was analyzed and adapted from the Hakulinen method.^26^^,^^27^ RS was estimated using the mortality tables for Khon Kaen Province. The results for RS were corrected for mortality by causes other than cancer, especially in older populations.

All statistical tests were two-sided with a significance level of 0.05. No adjustment of the alpha level was made for multiple testing. All statistical analyses were implemented using the Stata release 10 (StataCorp LLC, College Station, TX, USA).^28^

### Data processing

Data were recorded using CanReg 5 software provided by the IARC (International Agency for Research on Cancer, Lyon, France).^29^ The verification was performed with necessary correction, including logic, range, and internal consistency, which were checked using Stata 10.0 Statistical Software (Stata Corp)^28^ and Epidata Software (The EpiData Association, Denmark).^30^

### Ethical considerations

The present study was approved by the Institutional Review Board (HE581074), under the Office of Human Research Ethics, Khon Kaen University.

## RESULTS

### Descriptive epidemiology

We identified 11,711 cases of CCA in the KKCR database for the period 1989 through 2013. The male to female ratio was 2.2:1. The mean age was 62.6 (SD, 11.2) years. The age at diagnosis trended to be high. The other variables did not vary significantly. The most common stage of disease was ‘unknown staging’ (76.2%; *n* = 8,927) and “late stage” (ie, Stage III and IV; 23.3%; *n* = 2,722). Histological grading was commonly missing from the data (97.5%; *n* = 11,414) (Table 1). The primary bases of diagnosis were endoscopic and radiologic evidence versus morphological verification (10.6%; *n* = 1,247) (ie, based on either cytological or histological examination of tissue from the primary site, %MV) (data not shown).

**Table 1.** Characteristics of study participants at recruitment by 5-year periods

Characteristic	1989–1993		1994–1998		1999–2003		2004–2008		2009–2013	
				
	*n*	%	*n*	%	*n*	%	*n*	%	*n*	%
**1. Sex**										
Males	1,295	69.7	1,356	70.9	1,925	68.05	1,838	68.2	1,598	66.2
Females	564	30.3	557	29.1	904	31.95	857	31.8	817	33.8
Male to female ratio; 2.2:1	1.3:1		2.4:1		2.1:1		2.1:1		2.1:1	
**2. Age at diagnosis, year of age**										
30–40	104	5.6	75	4.0	94	3.3	62	2.3	34	1.4
41–50	300	16.2	291	15.3	344	12.2	281	10.5	172	7.1
51–60	630	33.9	611	31.9	754	26.7	737	27.4	532	22.0
61–98	825	44.3	936	48.8	1,637	57.8	1,615	59.8	1,677	69.5
Mean (SD) = 62.6 (11.2)	59.0 (10.9)		60.2 (10.8)		62.1 (10.9)		63.1 (10.8)		65.7 (10.6)	
Median (Min: Max) = 63 (30:98)	59.0 (30:92)		60.0 (30:94)		63.0 (30:97)		64.0 (30:96)		66.0 (30:98)	
**3. Stage at diagnosis**										
Stage I	—	—	5	0.3	—	—	1	—	4	0.2
Stage II	—	—	2	0.1	—	—	14	0.5	14	0.6
Stage III	3	0.2	—	—	2	0.1	15	0.6	41	1.7
Stage IV	446	24.0	394	20.6	560	19.8	654	24.5	607	25.1
Unknown	1,410	75.8	1,512	79.0	2,267	80.1	1,989	74.4	1,749	72.4
**4. Histological grading**										
Well-differentiated	—	—	11	0.6	61	2.2	54	2.0	52	2.2
Moderately-differentiated	—	—	1	0.1	21	0.7	22	0.8	24	1.0
Poorly-differentiated	1	0.1	—	—	20	0.7	14	0.5	12	0.5
Undifferentiated	—	—	—	—	1	—	2	0.1	1	—
Unknown	1,858	100.0	1,901	100.0	2,726	96.4	2,603	96.5	2,326	96.3

### Incidence

#### Age-standardized incidence rates (ASR)

**Figure 1.**
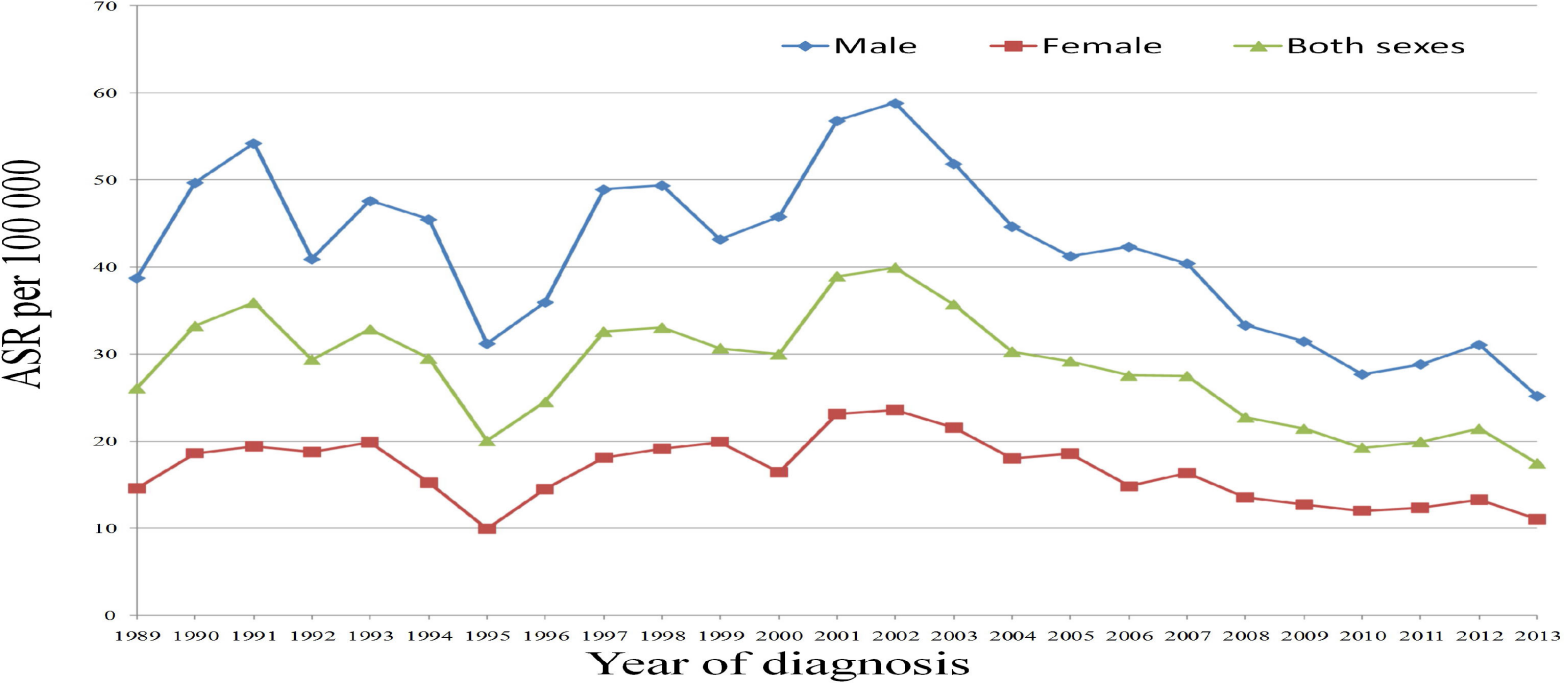
Incidence rates (per 100,000 per year) for CCA by sex in Khon Kaen Province from 1989 through 2013.

The respective ASR rate per 100,000 for CCA for (a) males, (b) females, and (c) males and females from 1989 through 2013 ranged from (a) 25.2 to 58.8, (b) 9.9 to 23.6, and (c) 17.5 to 39.9 (Figure 1). Thus, the overall ASR per 100,000 was 41.5 for males (95% CI, 40.6–42.4), 16.6 for females (95% CI, 16.1–17.1), and 28.1 for males and females (95% CI, 27.6–28.7) (Figure 1).

The ASR for CCA for all age groups, both males and females, for the whole period (1989 through 2013) has significantly decreased over time. The exception was for patients between 61 and 98 years, for whom the ASR initially increased in the first three periods but decreased in the last two (Table 2).

**Table 2.** Incidence by time period, age group, and sex in Khon Kaen Province from 1989 through 2013

Characteristic	Period of time	Males				Females				Both sexes			
			
Age group, years		*n*	CR	ASR	95% CI	*n*	CR	ASR	95% CI	*n*	CR	ASR	95% CI
30–40	1989–1993	53	1.23	1.1	0.77 to 1.34	25	0.61	0.5	0.30 to 0.69	78	0.96	0.77	0.6 to 0.95
	1994–1998	39	0.93	0.7	0.48 to 0.92	13	0.31	0.2	0.10 to 0.35	52	0.62	0.5	0.33 to 0.85
	1999–2003	52	1.22	0.9	0.64 to 1.12	17	0.39	0.3	0.15 to 0.41	69	0.80	0.6	0.44 to 0.71
	2004–2008	32	0.76	0.6	0.38 to 0.78	13	0.30	0.2	0.10 to 0.34	45	0.53	0.4	0.28 to 0.51
	2009–2013	21	0.50	0.4	0.24 to 0.60	5	0.11	0.1	0.01 to 0.18	26	0.30	0.3	0.15 to 0.35
41–50	1989–1993	191	4.4	5.2	4.44 to 5.92	82	2.0	2.2	1.72 to 2.68	273	3.67	3.7	3.23 to 4.11
	1994–1998	198	4.73	4.7	4.07 to 5.39	64	1.52	1.5	1.12 to 1.85	262	3.12	3.1	2.71 to 3.76
	1999–2003	212	4.96	4.3	3.74 to 4.91	87	2.01	1.8	1.40 to 2.15	299	3.47	3.0	2.68 to 3.37
	2004–2008	184	4.38	3.5	2.95 to 3.95	59	1.35	1.1	0.79 to 1.33	243	2.84	2.2	1.94 to 2.50
	2009–2013	111	2.66	1.9	1.58 to 2.31	40	0.93	0.6	0.44 to 0.83	151	1.97	1.4	1.22 to 1.65
51–60	1989–1993	429	12.52	16.1	14.73 to 17.45	163	3.98	4.5	3.85 to 5.24	592	7.26	8.6	7.87 to 9.25
	1994–1998	410	13.02	14.0	12.83 to 15.18	163	3.87	4.0	3.36 to 4.57	573	6.82	7.2	6.59 to 7.77
	1999–2003	519	12.14	11.5	10.47 to 12.44	200	4.61	4.2	3.61 to 4.76	719	6.65	6.2	5.65 to 6.66
	2004–2008	495	11.79	9.20	8.36 to 9.98	196	4.49	3.4	2.93 to 3.88	691	8.07	6.19	5.73 to 6.66
	2009–2013	331	7.92	5.2	4.66 to 5.79	146	3.28	2.1	1.78 to 2.47	477	5.53	3.6	3.29 to 3.94
61–98	1989–1993	614	14.23	23.4	21.4 to 25.34	293	7.16	11.1	9.84 to 12.40	907	11.13	18.5	17.32 to 19.74
	1994–1998	705	16.85	26.3	24.37 to 28.25	315	7.47	9.8	8.71 to 10.88	1020	12.4	17.3	16.25 to 18.37
	1999–2003	1136	21.61	28.5	26.62 to 30.28	598	13.78	14.9	13.70 to 16.09	1734	11.84	14.1	13.28 to 15.02
	2004–2008	1125	26.79	27.2	25.57 to 28.74	587	13.44	11.5	10.55 to 12.42	1712	19.99	18.5	17.67 to 19.43
	2009–2013	1134	27.13	21.3	20.9 to 22.58	626	14.08	9.4	8.69 to 10.19	1760	20.40	14.8	14.1 to 15.50

ASR, age-standardize rate; CI, confidence interval; CR, crude rate.

**Figure 2.**
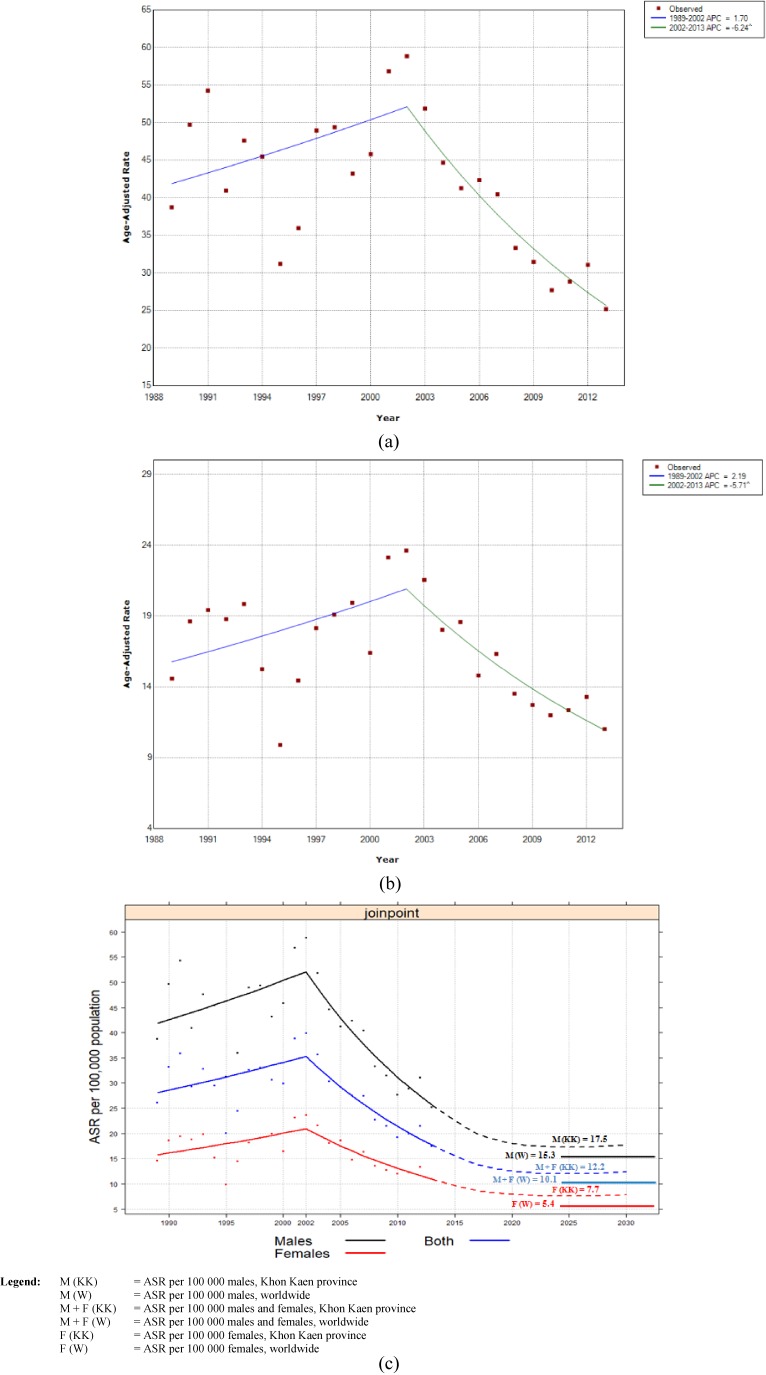
Joinpoint trends for age-adjusted rates per 100,000 for CCA in Khon Kaen Province from 1989–2002 and 2002–2013. a) Males; b) Females, and c) Simulated CCA incidence trend projections to 2030. ASR, age standardized rate.

Focusing on all ages, for the period 1989 through 2013, the Joinpoint regression revealed that the incidence was significantly decreasing by: (a) −2.0% per year among males (average annual percent change [AAPC] −2.0; 95% CI, −3.4 to −0.6); (b) −1.5% per year among females (AAPC −1.5; 95% CI, −3.1 to 0.1); and, (c) −1.9% per year among males and females (AAPC −1.9%; 95% CI, −3.3 to −0.5). Accoding to the Joinpoint analysis of years, the incidence rate among males increased with an APC of 1.7% (95% CI, −0.3 to 3.7) from 1989 through 2002, and decreased with an APC of 6.2% (95% CI, −8.4 to −4.0). By comparison, among females the incidence rate increased with an APC of 2.2% (95% CI, −0.2 to 4.6) from 1989 through 2002, and decresed with an APC of 5.7% (95% CI, −8.1 to −3.1) (Figure 2a and Figure 2b). The simulated projection curve of CCA incidence from 2014 through 2030 indicates that rates are expected to continue to decrease to 17.5 per 100,000 in males. Females are expected to reach 7.7 per 100,000, while both sexes are predicted to reach 12.2 per 100,000. The incidence will be stable over the next 10 years but will still exceed the average worldwide incidence (Figure 2c).

Lastly, for boths males and females, the incidence rate increased 1.8% (95% CI, −0.2 to 3.8) from 1989 through 2002, and decreased with an APC of 6.1% (95% CI, −8.2 to −3.9) (Table 3).

**Table 3.** Number of cases and annual percentage of change in incidence rate of CCA in Khon Kean Province from 1989 through 2013

Characteristic	Period of time	Males			Females			Both sexes		
			
All ages		*n*	APC	95% CI, *P*-value	*n*	APC	95% CI, *P*-value	*n*	APC	95% CI, *P*-value
	1989–2002	4,156	1.70	(−0.3 to 3.7), 0.100	1,823	2.19	(−0.2 to 4.6), 0.100	5,979	1.79	(−0.2 to 3.8), 0.100
	2002–2013	3,856	−6.24	(−8.4 to −4.0), <0.001	1,876	−5.71	(−8.1 to −3.2), <0.001	5,732	−6.09	(−8.2 to −3.9), <0.001

APC, annual percentage change; CI, confidence interval.

**Figure 3.**
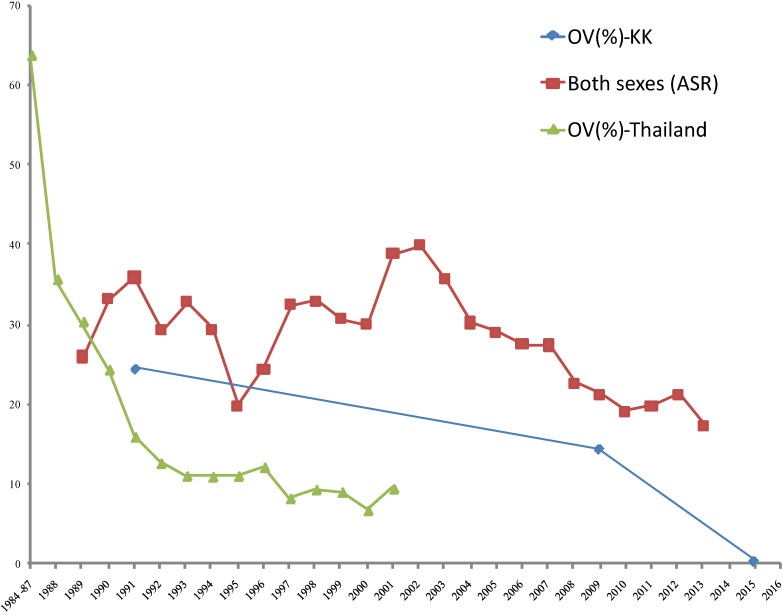
Age-adjusted rates per 100,000 (both sexes, ASR), OV (%)-Thailand, and OV (%)-KK in Khon Kaen Province trend downward. ASR, age standardized rate; KK, Khon Kaen Province; OV, *Opisthorchis viverrini*.

Figure 3 illustrates the incidence of CCA was declining parallel with the prevalence of *O. viverrini*. The data from the national and local level reveal a decreasing proportion of infection, which is consistent with the ASR for CCA in Thailand.

### Survival

The respective OS versus RS at 1, 3, and 5 years for the different age groups of CCA is presented in Table 4. At 5 years, the respective OS and RS for males aged 30–40, 41–50, 51–60, and 61–98 years of age was 23.2% (95% CI, 17.0 to 30.4%) versus 23.4% (95% CI, 17.1 to 31.6%), 12.6% (95% CI, 10.1 to 15.7%) versus 13.1% (95% CI, 10.5 to 16.2%), 7.4% (95% CI, 6.0 to 9.0%) versus 7.7% (95% CI, 6.2 to 9.2%), and 6.8% (95% CI, 5.9 to 7.8%) versus 6.9% (95% CI, 6.0 to 7.9%). For females, the respective OS versus RS for age groups of 30–40, 41–50, 51–60, and 61–98 years of age was 19.0% (95% CI, 10.2 to 35.4%) versus 19.2% (95% CI, 10.3 to 35.7%), 16.7% (95% CI, 12.3 to 22.5%) versus 17.5% (95% CI, 13.2 to 23.4%), 11.1% (95% CI, 8.7 to 14.1%) versus 11.2% (95% CI, 8.8 to 14.2%), and 7.6% (95% CI, 6.4 to 9.1%) versus 7.7% (95% CI, 6.4 to 9.2%) (Table 4).

**Table 4.** Overall observed survival and relative survival of CCA for each age-group and sex in Khon Kaen Province from 1989 through 2013

Characteristic	Survival time	Males			Females			Both sexes		
			
Age groups, years		*n*	OS (95% CI)	RS (95% CI)	*n*	OS (95% CI)	RS (95% CI)	*n*	OS (95% CI)	RS (95% CI)
30–40	1 year	49	35.5 (28.5 to 44.1)	36.3 (29.3 to 45.1)	19	38.7 (27.6 to 54.3)	41.0 (29.6 to 56.6)	68	37.0 (30.9 to 44.3)	37.7 (31.5 to 45.0)
	3 years	28	25.2 (18.8 to 33.6)	26.4 (19.9 to 34.9)	11	24.9 (15.2 to 40.8)	25.1 (15.3 to 41.2)	39	25.1 (20.2 to 33.7)	26.1 (20.4 to 33.3)
	5 years	23	23.2 (17.0 to 30.4)	23.4 (17.2 to 31.6)	7	19.0 (10.2 to 35.4)	19.2 (10.3 to 35.7)	25	22 (16.6 to 29.2)	22.3 (16.8 to 29.5)
41–50	1 year	141	24.8 (21.6 to 28.4)	25.1 (21.9 to 28.7)	61	26.6 (21.5 to 32.9)	27.6 (22.4 to 34.0)	202	25.5 (22.8 to 28.6)	25.7 (23.0 to 28.8)
	3 years	68	13.8 (11.3 to 17.0)	13.9 (11.4 to 17.1)	37	18.0 (13.6 to 23.8)	18.1 (13.7 to 24.0)	105	14.9 (12.6 to 17.7)	15.1 (12.8 to 17.8)
	5 years	40	12.6 (10.1 to 15.7)	13.1 (10.5 to 16.2)	23	16.7 (12.3 to 22.5)	17.5 (13.2 to 23.4)	63	13.7 (11.4 to 16.4)	14.3 (12.0 to 17.0)
51–60	1 year	284	19.3 (17.4 to 21.3)	19.4 (17.6 to 21.5)	135	23.1 (20.0 to 26.6)	23.3 (20.2 to 26.8)	419	20.4 (18.8 to 22.2)	20.5 (18.9 to 22.3)
	3 years	108	9.2 (7.9 to 10.9)	9.4 (8.0 to 11.0)	62	13.3 (10.8 to 16.4)	13.6 (11.1 to 16.7)	170	10.4 (9.1 to 11.8)	10.6 (9.3 to 12.0)
	5 years	46	7.4 (6.0 to 9.0)	7.6 (6.2 to 9.2)	40	11.1 (8.7 to 14.1)	11.2 (8.8 to 14.2)	86	8.5 (7.3 to 9.9)	8.6 (7.8 to 10.0)
61–98	1 year	534	16.8 (15.6 to 18.1)	16.8 (15.6 to 18.1)	329	19.4 (17.7 to 21.3)	19.5 (17.7 to 21.4)	863	17.7 (16.7 to 18.7)	17.7 (16.7 to 18.8)
	3 years	227	8.6 (7.7 to 9.7)	8.7 (7.8 to 9.8)	125	9.8 (8.4 to 11.3)	9.9 (8.5 to 11.5)	352	9.1 (8.3 to 9.9)	9.1 (8.3 to 10.0)
	5 years	114	6.8 (5.9 to 7.8)	6.9 (6.0 to 7.9)	64	7.6 (6.4 to 9.1)	7.7 (6.4 to 9.2)	174	7.1 (6.4 to 7.9)	7.2 (6.4 to 8.0)

CI, confidence interval; OS, overall survival; RS, relative survival.

**Figure 4.**
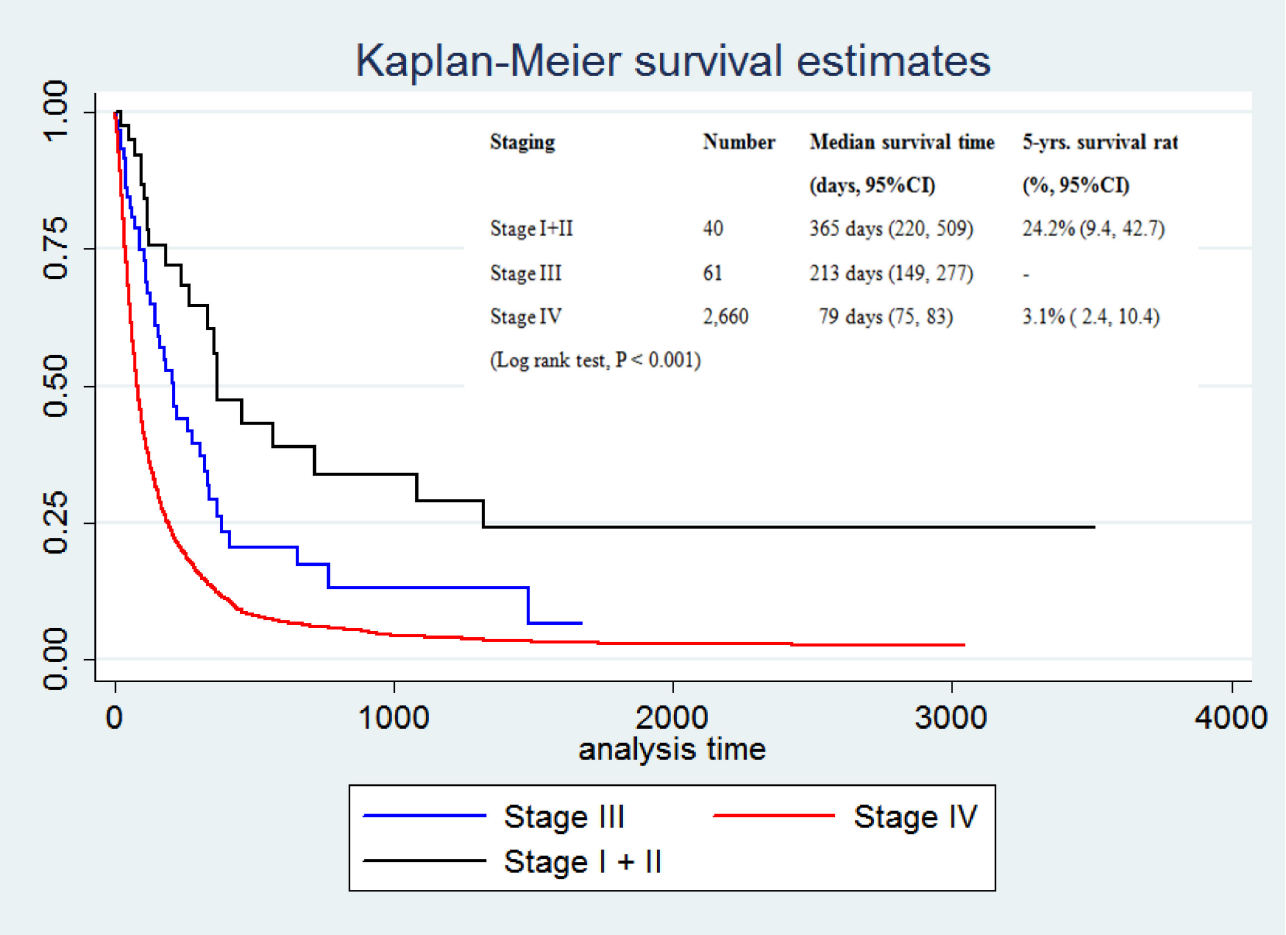
Kaplan-Meier survival curves for patients with CCA in Khon Kaen Province from 1989 through 2013. The curves represent the TMN stage.

**Figure 5.**
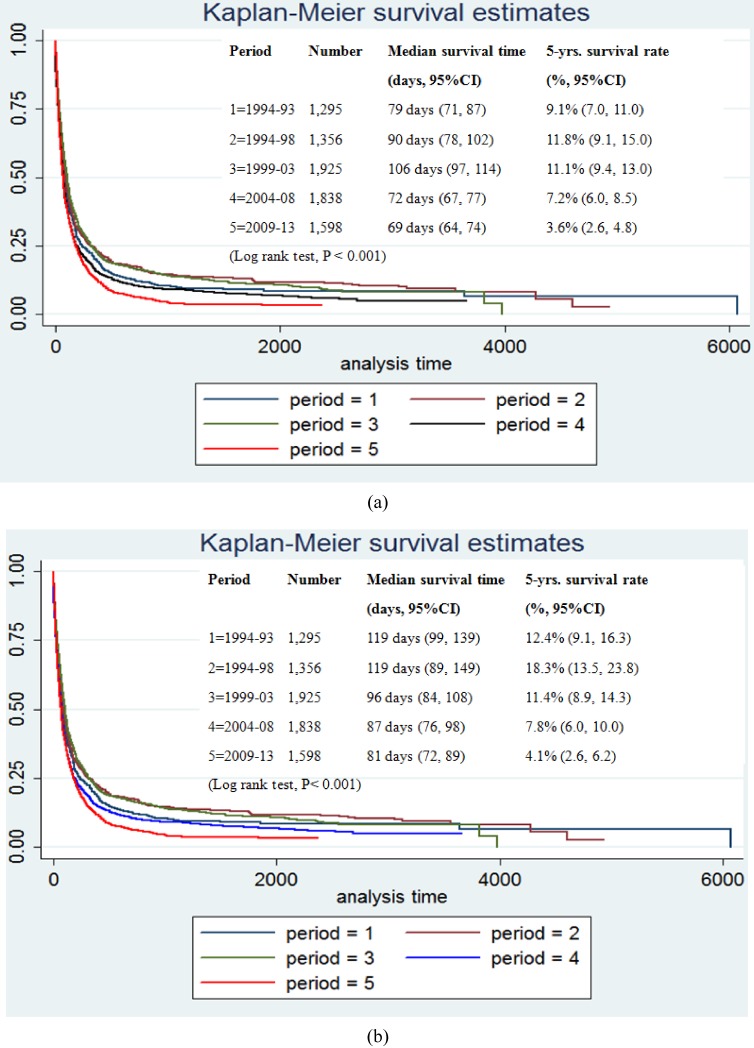
Kaplan-Meier survival curves for males (a) and females (b) with CCA in Khon Kaen Province from 1989 through 2013. The curves represents periods of time. CI, confidence interval.

Focusing on stage of disease and period of time, the results of the log-rank test showed significant relationships between patient survival and stage of disease and period of time (*P*-value <0.001) (Figure 4 and Figure 5).

## DISCUSSION

The current study showed that the incidence of CCA has been significantly decreasing by (a) −2.0% per year among males, (b) −1.50% per year among females, and (c) −1.9 per year among males and females. This is consistent with the reported incidence in our previous study and the ASR of liver and bile duct cancer as reported by the Thailand Cancer Registry. The most common histological type was CCA.^2^^–^^9^

We have hypothesized that the decline in the incidence of CCA in our previous study may be the real falling risk.^15^ The updated decline in incidence confirms our hypothesis. The decrease in the incidence of CCA might be the result of controlling the risk factors associated with *O. viverrini* infection. The incidence of *O. viverrini* infection has been decreasing over time, from >60% in 1984 to <10% after 1997.^31^ The declining incidence parallels a decline in *O. viverrini* infection rates over the last 20 years.

Several studies have addressed the unique risk factors of CCA in some countries.^32^ Since *O. viverrini* infection is believed to be one of the risk factors of cholangiocarcinogenesis in Thailand,^33^ a process that takes decades, time was also needed to evaluate the effectiveness of *O. viverrini* infection control (Figure 3). Numerous government policies aimed at decreasing the rate of *O. viverrini* infection, including: (a) liver fluke control units, established in 1967; (b) continuous health education, also established in 1967; (c) a liver fluke control program, embedded in the 5-year National Public Health Development Plan (1987–1991)^34^; and (d) the Promotion of Community Health through Parasitic Control in seven northeastern provinces, in cooperation with the Federal Republic of Germany government, run from 1989–1992. The liver fluke control program continues to be an element of the National Public Health Developmant Plan.^14^^,^^31^

Our data show that, while the elderly continue to eat raw fish, the younger generations are avoiding eating it. The results indicate that *O. viverrini* infection occurred predominantly in the elderly over younger age groups.^35^ Education appears to be more effective in the young than the elderly. The life cycle of *O. viverrini* and risk factors for CCA should thus be introduced in primary school.

We found that the predicted incidence of CCA will be stable over the next 10 years, albeit higher than in other parts of the world,^36^ suggesting that there are unidentified risk factors other than *O. viverrini* infection and nitrosamine. With respect to the latter factor, in other research, we attempted to demonstrate that repeated use of praziquantel could increase the risk of CCA, but the evidence was weak.^37^ Further research is needed to identify other risk factors for CCA, particularly other environmental factors that could be controlled (ie, pesticides and carcinogens in the food chain).

The survival of CCA patients remains poor, despite improvements in diagnostic technology and surgical techniques. Several reasons may account for this finding: (a) most patients in northeastern Thailand present for care at a late stage of CCA, when only palliative treatment is an option^38^; (b) stringent criteria for resection means that some patients are denied surgery; and (c) after 2006, a new classification of bile duct tumors (ie, intraductal papillary neoplasm of the bile duct [IPNB]) was introduced, which was previously included with CCA. Since the prognosis of IPNB is relatively good, the survival of CCA before 2006 appeared to have been better than after IPNB was no longer included as a CCA.^39^^,^^40^

### Conclusion

The incidence of CCA in Khon Kaen Province has been decreasing over the last 10–12 years, coinciding with government efforts to control risk factors related to *O. viverrini* infection. The projected incidence of CCA should remain stable over the next 10 years, although it is higher than the worldwide incidence. The survival of CCA patients remains poor, so it is important to identify other risk factors, apart from *O. viverrini* and nitrosamine, that could be targeted to lower the incidence, as well as employing a screening program to detect eartlier stage that might improve survival.
