# Cholangiocarcinoma Trends, Incidence, and Relative Survival in Khon Kaen, Thailand From 1989 Through 2013: A Population-Based Cancer Registry Study

**DOI:** 10.2188/jea.JE20190251

**Published:** 2020-02-05

**Authors:** Supot Kamsa-ard, Vor Luvira, Krittika Suwanrungruang, Siriporn Kamsa-ard, Varisara Luvira, Chalongpon Santong, Tharatip Srisuk, Ake Pugkhem, Vajarabhongsa Bhudhisawasdi, Chawalit Pairojkul

**Affiliations:** 1 Department of Epidemiology and Biostatistics, Faculty of Public Health, Khon Kaen University, Khon Kaen, Thailand; 2 ASEAN Cancer Epidemiology and Prevention Research Group, Khon Kaen University, Khon Kaen, Thailand; 3 Department of Surgery, Faculty of Medicine, Khon Kaen University, Khon Kaen, Thailand; 4 Cancer Unit, Srinagarind Hospital, Faculty of Medicine, Khon Kaen University, Khon Kaen, Thailand; 5 Department of Community Medicine, Faculty of Medicine, Khon Kaen University, Khon Kaen, Thailand; 6 Department of Pathology, Faculty of Medicine, Khon Kaen University, Khon Kaen, Thailand

There were three errors in **Figure 5**, and we have corrected data in the result sections. First, in the results section (page 203, **Figure 5A**), there is an error in the legend box. (Column 1 as “Period”), the label “1” should be amended a “1=1989–93”. It is not, “1=1994–93”

Second, in the results section (page 203, **Figure 5B**), there are errors in the legend box (Column 1 as “Period”), the label “1” should be amended a “1=1989–93”. It is not, “1=1994–93”. The column 2 as “Number”, the number for “1=1989–93”, “2=1994–98”, “3=1999–03”, “4=2004–08”, “5=2009–13” should be amended as 564, 557, 904, 857, and 817, respectively.

Third, in the results section (page 203, **Figure 5B**), the Survival curve was duplicated from **Figure 5A**, it should be changed to the correct figure.

The correct **Figure 5** is provided below. The authors apologize for these errors.

**Figure 5.**
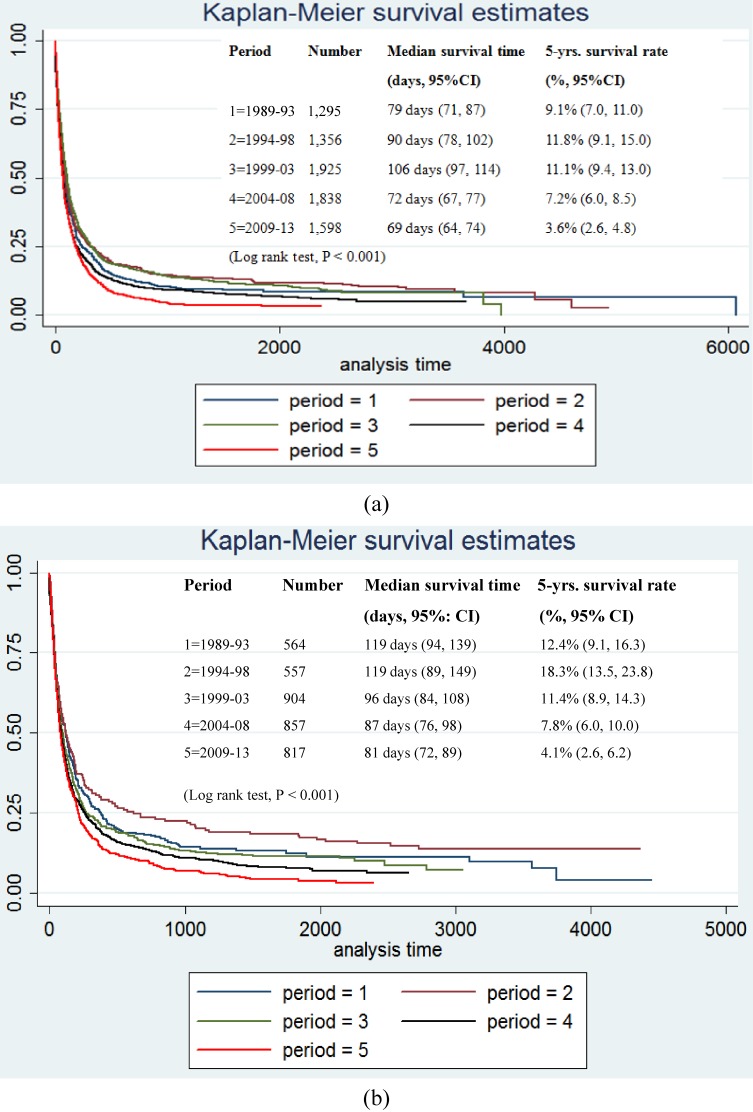
Kaplan-Meier survival curves for males (a) and females (b) with CCA in Khon Kaen Province from 1989 through 2013. The curves represents periods of time. CI, confidence interval.

